# Supplementary figures and images for: Anomalous diffusion for neuronal growth on surfaces with controlled geometries
Source: PLoS One. 2019 May 6;14(5):e0216181. doi: 10.1371/journal.pone.0216181 (PMC6502317; doi:10.1371/journal.pone.0216181)

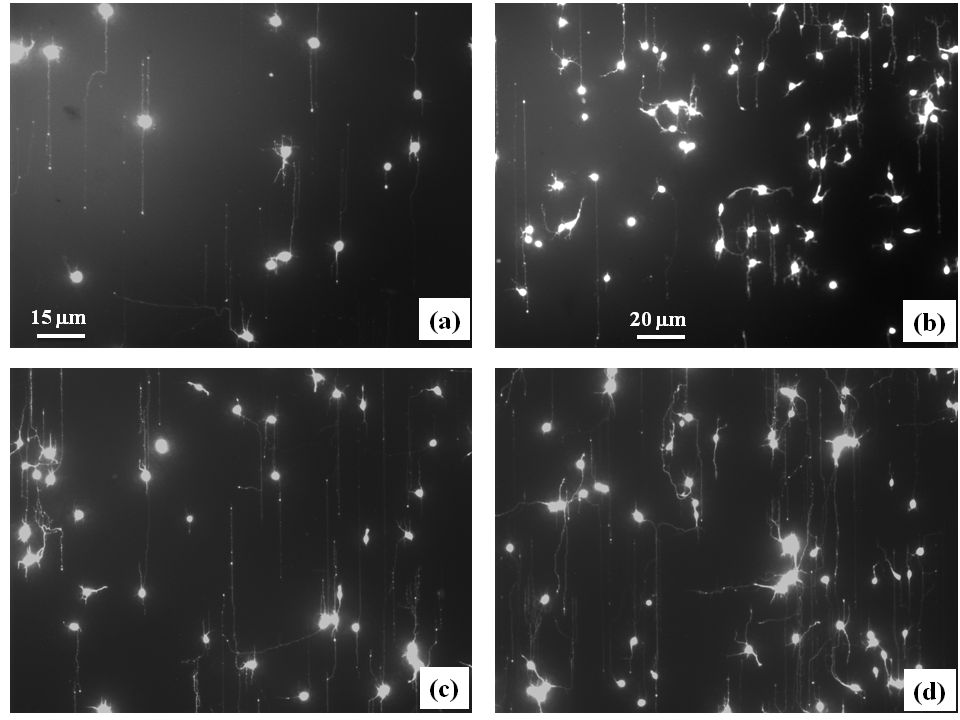

Supplement: S1 Fig — (a) Neurons imaged at t = 16 hrs after plating. (b) Neurons imaged at t = 32 hrs after plating. (c) Neurons imaged at t = 64 hrs after plating. (d) Neurons imaged at t = 80 hrs after plating. The scale bar is 15 μm in (a) and 20 μm in (b-d). (TIF) [file pone.0216181.s001.tif]

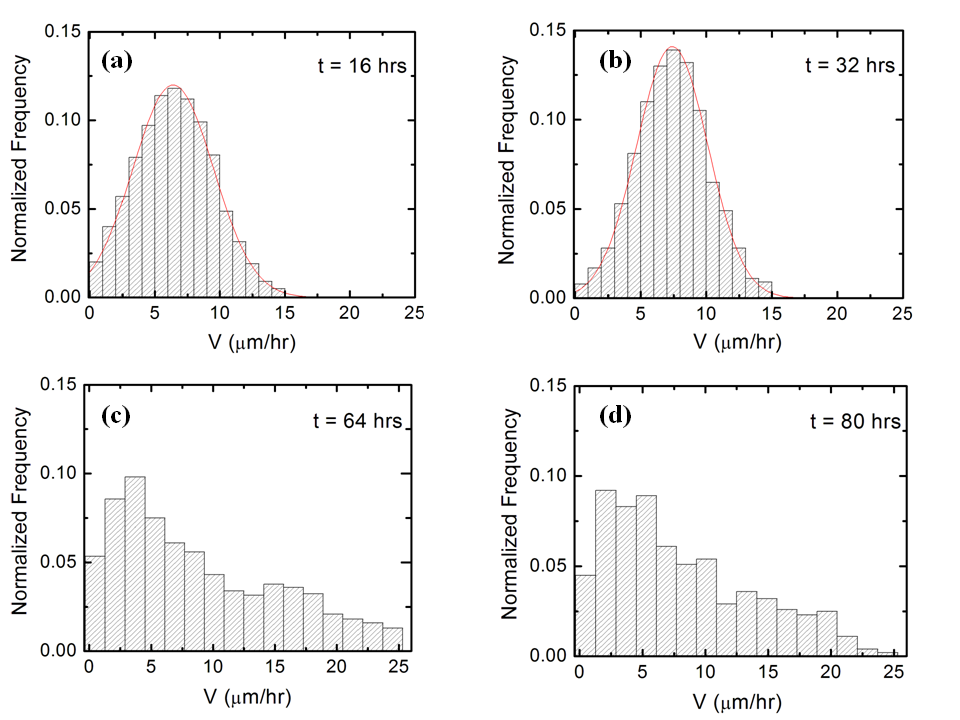

Supplement: S2 Fig — (a) Speed distribution for N = 182 different growth cones, measured at t = 16 hrs after plating. The continuous red curve represents fit with the Gaussian distribution given by Eq 6. (b) Speed distribution for N = 195 different growth cones measured at t = 32 hrs after plating. The continuous red curve represents fit with the Gaussian distribution given by Eq 6. (c) Speed distribution for N = 179 different growth cones measured at t = 64 hrs after plating. (d) Speed distribution for N = 168 different growth cones measured at t = 80 hrs after plating. (TIF) [file pone.0216181.s002.tif]

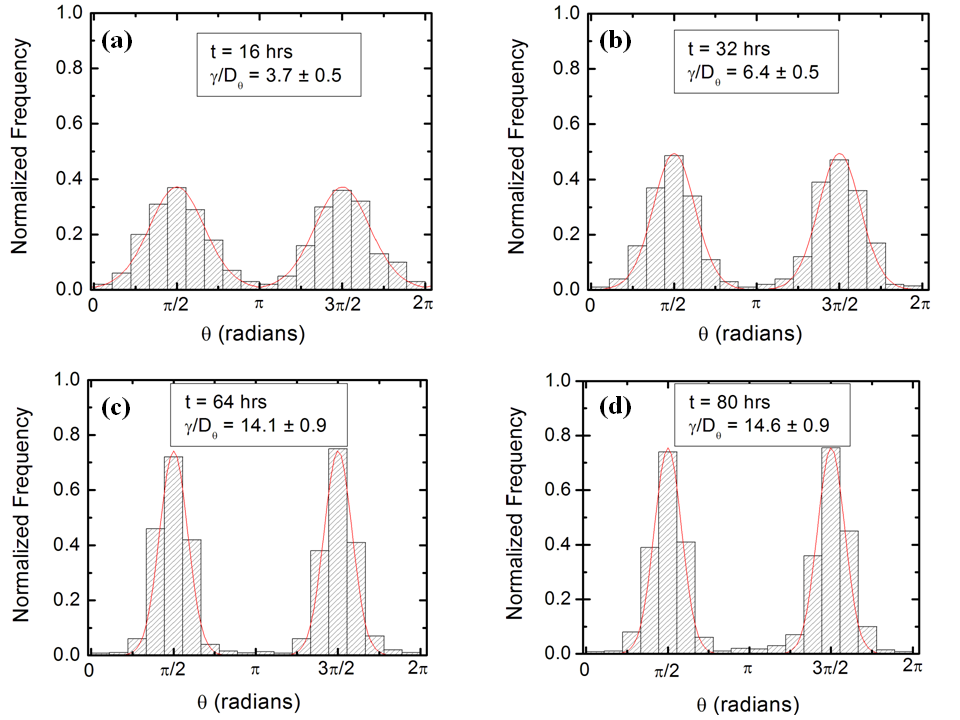

Supplement: S3 Fig — The vertical axis (labeled Normalized Frequency) represents the ratio between the number of axonal segments growing in a given direction and the total number N of axon segments measured at a given time point t. Each axonal segment is of 20 μm in length (see Data Analysis section). (a) Data for N = 1877 different axon segments obtained at t = 16 hrs after plating. (b) Data for N = 2383 different axon segments obtained at t = 32 hrs after plating. (c) Data for N = 2537 different axon segments obtained at t = 64 hrs after plating. (d) Data for N = 2903 different axon segments obtained at t = 80 hrs after plating. The axons display strong directional alignment along the surface patterns (peaks at θ = π/2 and θ = 3π/2), with the degree of alignment (sharpness of the distribution) increasing with time. The continuous red curves in each figure represents fit to the data points using Eq 14. The data fit gives the ratio γθ/Dθ between the deterministic torque and the diffusion coefficient for the angular motion, at each time point (see text). (TIF) [file pone.0216181.s003.tif]

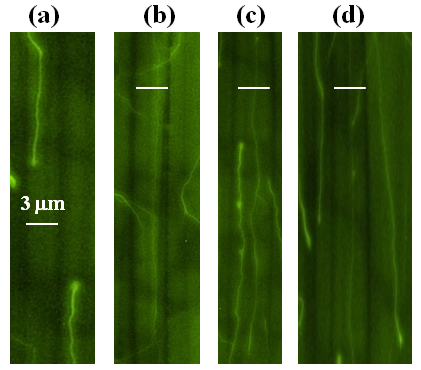

Supplement: S4 Fig — The images have been taken using the high magnification objective (60x) of the Nikon Eclipse Ti microscope, at different locations on 2 different substrates. The images show the fluorescently labeled microtubules (green), i.e. the C domain (see ref. [1]) inside the axons. The microtubules are labeled using Tubulin Tracker Green (see main text). The position of the micro-patterned troughs is shown by the vertical black lines. The 3μm white scale bar shows the distance between two adjacent troughs, and it has the same size for all images. The images show that the axons are located on the ridges of the patterns. The position of the ridges and troughs has been verified using AFM (images similar to the one shown in Fig 2). (TIF) [file pone.0216181.s004.tif]

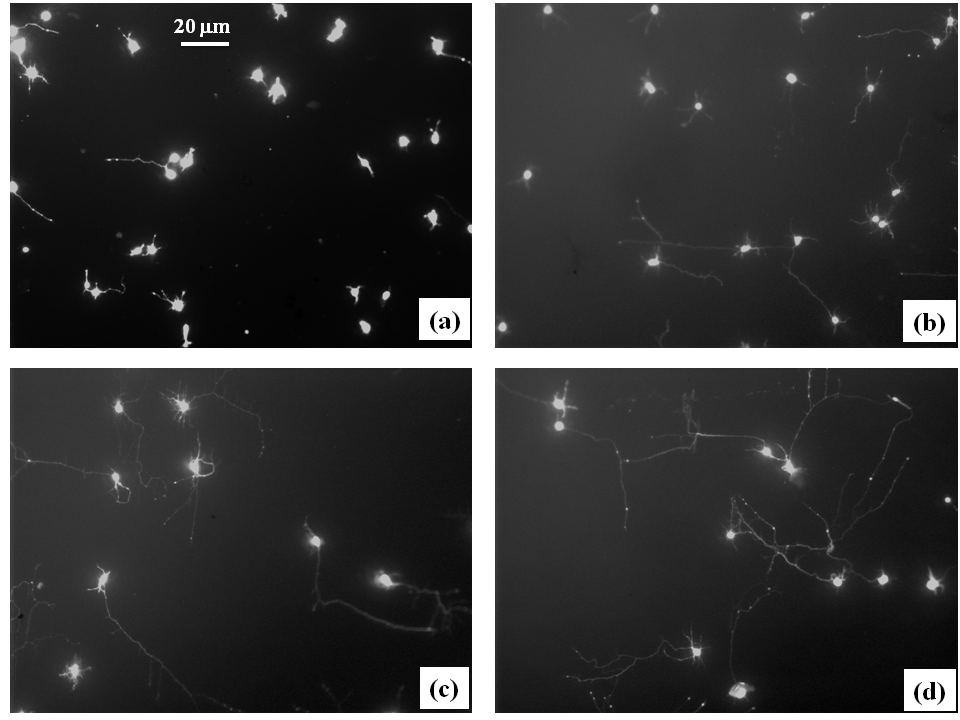

Supplement: S5 Fig — (a) Neurons imaged at t = 6 hrs after plating. (b) Neurons imaged at t = 24 hrs after plating. (c) Neurons imaged at t = 48 hrs after plating. (d) Neurons imaged at t = 72 hrs after plating. The scale bar is 20 μm in all images. (TIF) [file pone.0216181.s005.tif]

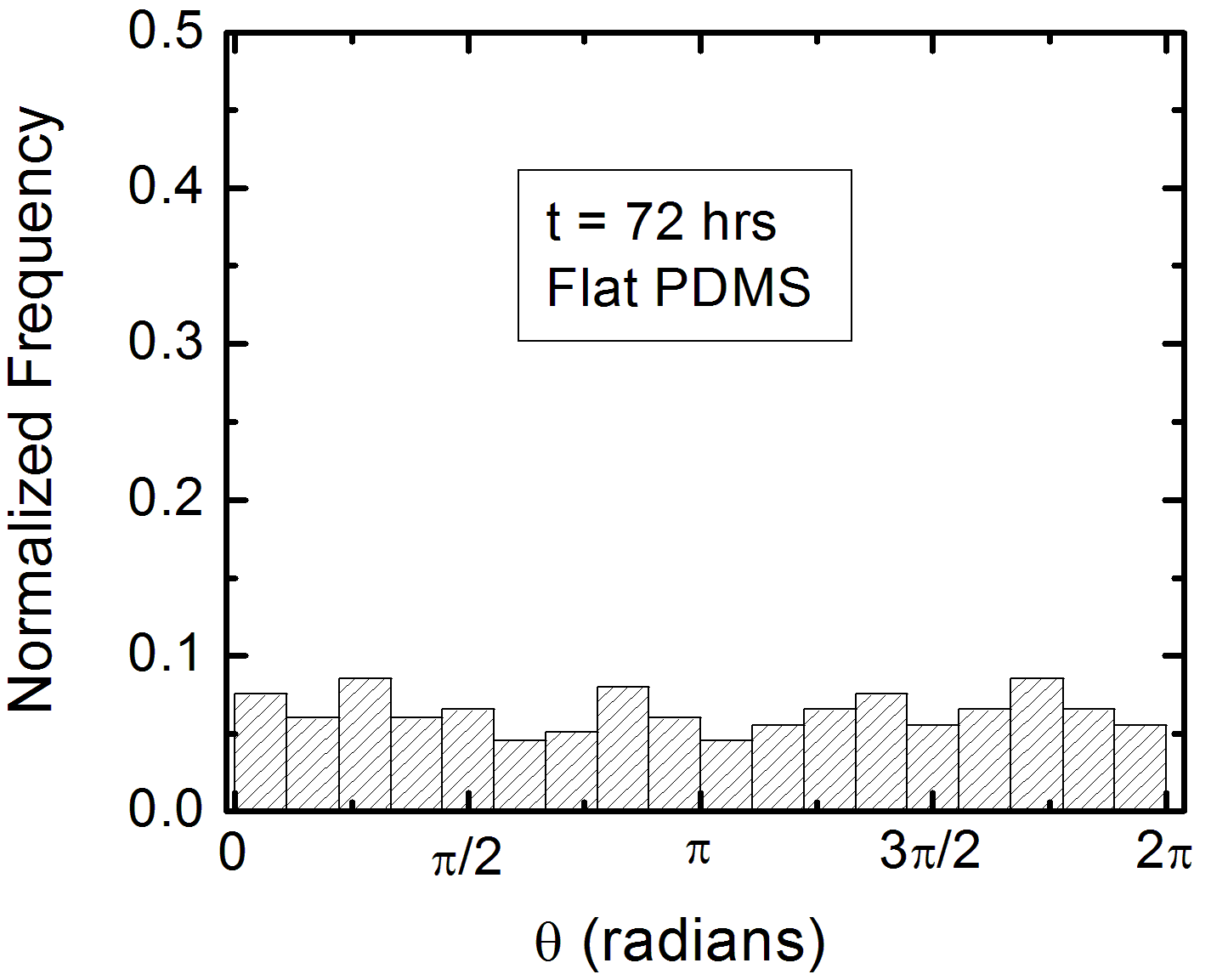

Supplement: S6 Fig — The vertical axis (labeled Normalized Frequency) represents the ratio between the number of axonal segments growing in a given direction and the total number N of axon segments measured. Each axonal segment is of 20 μm in length (see Data Analysis section). The data was taken for N = 1020 different axon segments measured at t = 72 hrs after plating. The angular distribution demonstrates that there is no axonal alignment, in contrast to the case of neuronal growth on patterned PDMS (Fig 5 and S3 Fig). (TIF) [file pone.0216181.s006.tif]

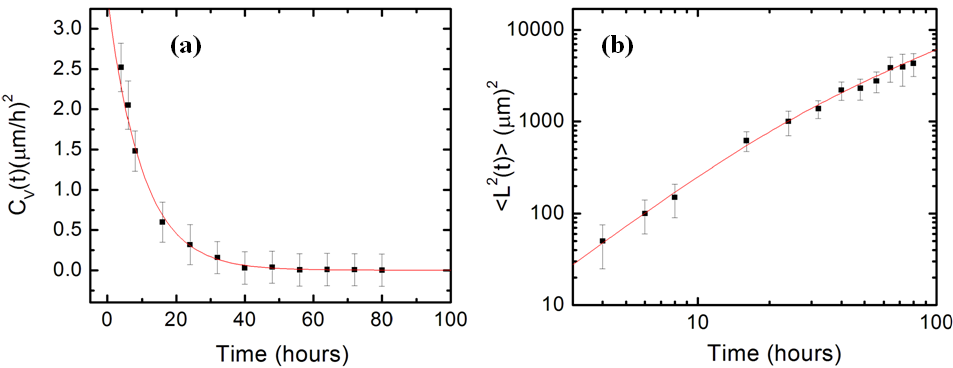

Supplement: S7 Fig — (a) Data points: experimentally measured velocity autocorrelation function vs. time. The continuous red curve represents the fit of the data points with the prediction of the theoretical model based on the Ornstein-Uhlenbeck process (Eq 7). (b) log-log plot of axonal mean square length vs. time. The continuous red curve represents the fit to the data with Eq 8 (prediction of the theoretical model based on the Ornstein-Uhlenbeck process). Each data point in (a) and (b) was obtained by measuring between N = 70 and N = 137 different axons (corresponding to 3–6 different fluorescent images per time data point). Error bars in both figures indicate the standard error of the mean. The fit of the data in with Eq 7 for (a), and Eq 8 for (b) give the diffusion coefficient D and the constant damping coefficient γ of the Ornstein-Uhlenbeck process (see text). (TIF) [file pone.0216181.s007.tif]
